# Building resilient medical technology supply chains with a software bill of materials

**DOI:** 10.1038/s41746-021-00403-w

**Published:** 2021-02-23

**Authors:** Seth Carmody, Andrea Coravos, Ginny Fahs, Audra Hatch, Janine Medina, Beau Woods, Joshua Corman

**Affiliations:** 1 MedCrypt, San Diego, CA USA; 2 DRX Labs LLC, Monrovia, MD USA; 3 Elektra Labs, Inc, San Francisco, CA USA; 4 Harvard-MIT Center for Regulatory Science, Boston, MA USA; 5 Biohacking Village, Las Vegas, NV USA; 6 Pennsylvania State University Policy Innovation Lab of Tomorrow (PILOT), State College, PA USA; 7 Aspen Institute Tech Policy Hub, San Francisco, CA USA; 8 I Am The Cavalry, Washington, DC USA; 9 Thermo Fisher Scientific, Inc, Waltham, MA USA; 10 US Department of Defense Technology Transfer Program, New York, NY USA; 11 Atlantic Council, Washington, DC USA; 12 Heinz College of Information Systems and Public Policy, Carnegie Mellon University, Pittsburgh, PA USA; 13 PTC, Inc, Boston, MA USA

**Keywords:** Technology, Industry

## Abstract

An exploited vulnerability in a single software component of healthcare technology can affect patient care. The risk of including third-party software components in healthcare technologies can be managed, in part, by leveraging a software bill of materials (SBOM). Analogous to an ingredients list on food packaging, an SBOM is a list of all included software components. SBOMs provide a transparency mechanism for securing software product supply chains by enabling faster identification and remediation of vulnerabilities, towards the goal of reducing the feasibility of attacks. SBOMs have the potential to benefit all supply chain stakeholders of medical technologies without significantly increasing software production costs. Increasing transparency unlocks and enables trustworthy, resilient, and safer healthcare technologies for all.

Cybersecurity is a national security issue. Healthcare public health was identified by the Presidential Policy Directive 21 (PPD-21) as one of sixteen critical infrastructure sectors^1^ and has a significantly large environment open to unauthorized attacks, also referred to as an “attack surface”. The 2009 Health Information Technology for Economic and Clinical Health (HITECH) Act^2^ incentivized the connection of and conversion from isolated, disparate, often paper-based systems to electronic medical records to improve public health outcomes. Other connected healthcare technologies, such as bedside monitors for cardiac implants, have already improved patient outcomes^3^.

Connectivity of medical devices and systems increases patient benefits. Yet, connectivity also broadens the exposure of vulnerabilities across systems within the healthcare supply chain that, if exploited, can compromise healthcare delivery, thereby increasing patient risk (see Fig. 1)^4^. For example, the WannaCry ransomware attack impacted healthcare delivery in over a third of the United Kingdom’s National Health System (NHS) trusts^5,6^. In another example, the NotPetya cyberattack rendered unnamed products unavailable by disrupting manufacturing, research, and sales operations of Merck^7^. Other industries were also disrupted, including Maersk’s global shipping operations^8,9^.

**Fig. 1:**
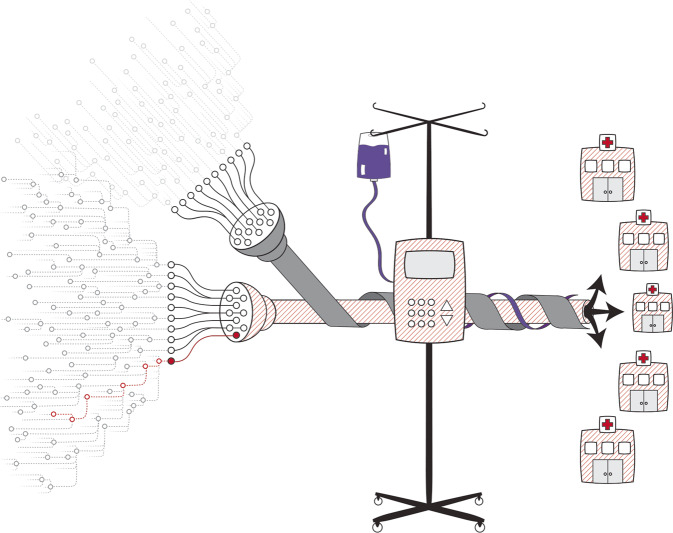
The impact of vulnerabilities. A single vulnerability in a single third-party component has the potential to impact individual or classes of devices across innumerable healthcare organizations. Reprinted from NTIA Use Cases and State of Practice Working Group^4^.

This connectivity, with its benefits and risks, has been facilitated by software. In general, the reuse of software components, such as third-party software components, has been an effective way of reducing costs and the time and resources required during the software development cycle^10^. A 2017 audit and analysis of over 1100 commercial, cross-sector codebases found that 96% of software products^11^ included third-party software components such as commercial off-the-shelf components, modules, and libraries from both open-source and commercial third-party suppliers^12^. Reliance on third-party components to deliver needed functionality carries with it the potential for increased risk. For example, a single vulnerability in a third-party component upstream can potentially have profound downstream impacts on patient health, privacy, and safety.

Vulnerabilities in common third-party components can—and have—greatly impacted delivery of patient care. For instance, the WannaCry attack in May 2017 infected 200,000 computers in hospital systems across 150 countries^13^. These exploits leveraged a vulnerability in several versions of Microsoft Windows for which a patch had been issued in March 2017^14^, 2 months prior to the attack. In the absence of a published software bill of materials (SBOM), builders such as medical device manufacturers and operators such as healthcare delivery organizations (HDOs) likely would have had to manually inventory systems to detect the vulnerable software versions. These resource-intensive processes can contribute to delays in patch validation, patch installation, and consequently, inoculation of systems. In another instance, vulnerabilities in JBoss—an open source technology library—led to critical outages at several HDOs despite the availability of updates for as long as 10 years in some cases^15^. Medical devices themselves can have thousands of vulnerabilities from third-party software components^16–18^, including approximately 1% of UK NHS devices impacted by WannaCry^5^. While SBOMs are not a panacea for cybersecurity, they can be effective (i.e., timely) for cybersecurity risk management. In each of these cases, had a preidentified list of third-party software components been accessible to customers such as HDOs, risk mitigation measures may have been pre-positioned and resources could have been efficiently targeted toward only affected, high-risk systems, thereby enabling more rapid incident response and potentially reducing disruption to healthcare delivery on a global scale.

## A history of the software bill of materials

W. Edwards Deming, often credited with inspiring Japan’s post-war economic boom and the rise of manufacturing paradigms such as Toyota’s Supply Chain Management^19,20^, used the concept of a bill of materials (BOM) to track the parts used to create a product. The idea was that if defects are found in a specific part, manufacturers can use the BOM to easily locate affected products^21^. Tracking the provenance of parts across the supply chain also allows manufacturers to improve the quality of suppliers they select.

An SBOM is analogous to the list of ingredients on food packaging^21^. The ingredients list provides transparency about components (e.g., salt, nuts, and high-fructose corn syrup), allowing individuals with medical conditions, allergies, or preferences to make better buying decisions. Software engineers build products by assembling open-source and commercial software “components”, which are smaller pieces of software built by third parties. Similar to an ingredients list, an SBOM lists every component of software in the finished product. This ensures that anyone who chooses the product knows its relative hygiene, and anyone who uses the software knows what is inside. When a widespread vulnerability is discovered, SBOMs enable patients or organizations such as HDOs to identify impacted technology that might be in use.

The BOM concept has been applied to software supply chains for configuration management^22^, and working models pertaining to software updates, emergency management, and software licensing have existed since the late 1990s^23–25^. Recently, from a security perspective, SBOMs have been conceptually applied towards supply-chain assurance^26,27^, including as a potential solution for healthcare security risk^28^.

An example of effective application of the SBOM concept comes from the financial services industry. By 2015, a series of software supply-chain vulnerabilities had forced the industry to re-evaluate the third-party software in its infrastructure^29^. This sector quickly adopted SBOM concepts into their internal development and procurement processes^30–33^. Now, if a vendor can provide an SBOM, it serves as a litmus test for the maturity of the vendor’s organization. If vendors lack an SBOM, many financial services organizations anticipate that their products will likely cost more to evaluate, operate, and own over their lifecycles. As a result, the financial organizations might negotiate discounts to account for these increased costs^32,33^.

The energy sector likewise has adopted SBOM procedures to reduce vulnerabilities. In 2014, the Energy Sector Control Systems Working Group (ESCSWG) and its collaborators published standardized procurement language^34^. This “toolkit” aims to reduce cybersecurity risk by managing known vulnerabilities and delivering more secure systems. The software section of this document offers users specific language to include in contracts, which would require vendors to provide documentation of all components of the product, plans for their maintenance, and protocols for reducing various types of risk throughout the product’s lifecycle.

Although mounting security problems in healthcare and their root causes have clarified that SBOMs might solve several problems, implementation has been slow and there are few data available from the published peer-reviewed literature. Complicating this issue is a lack of out-of-the-box solutions and industry-wide standards, such that organizations have developed homegrown proprietary solutions to improve interoperability and security of their systems. As one example, the Mayo Clinic now requires prospective vendors of medical devices to submit a complete description of all components of their products, including software architecture, as part of its procurement process^35^. This is a rare instance of such information being publicly available for a healthcare entity, however.

## The role of software bill of materials in proactive risk mitigation and resilience

**Fig. 2:**
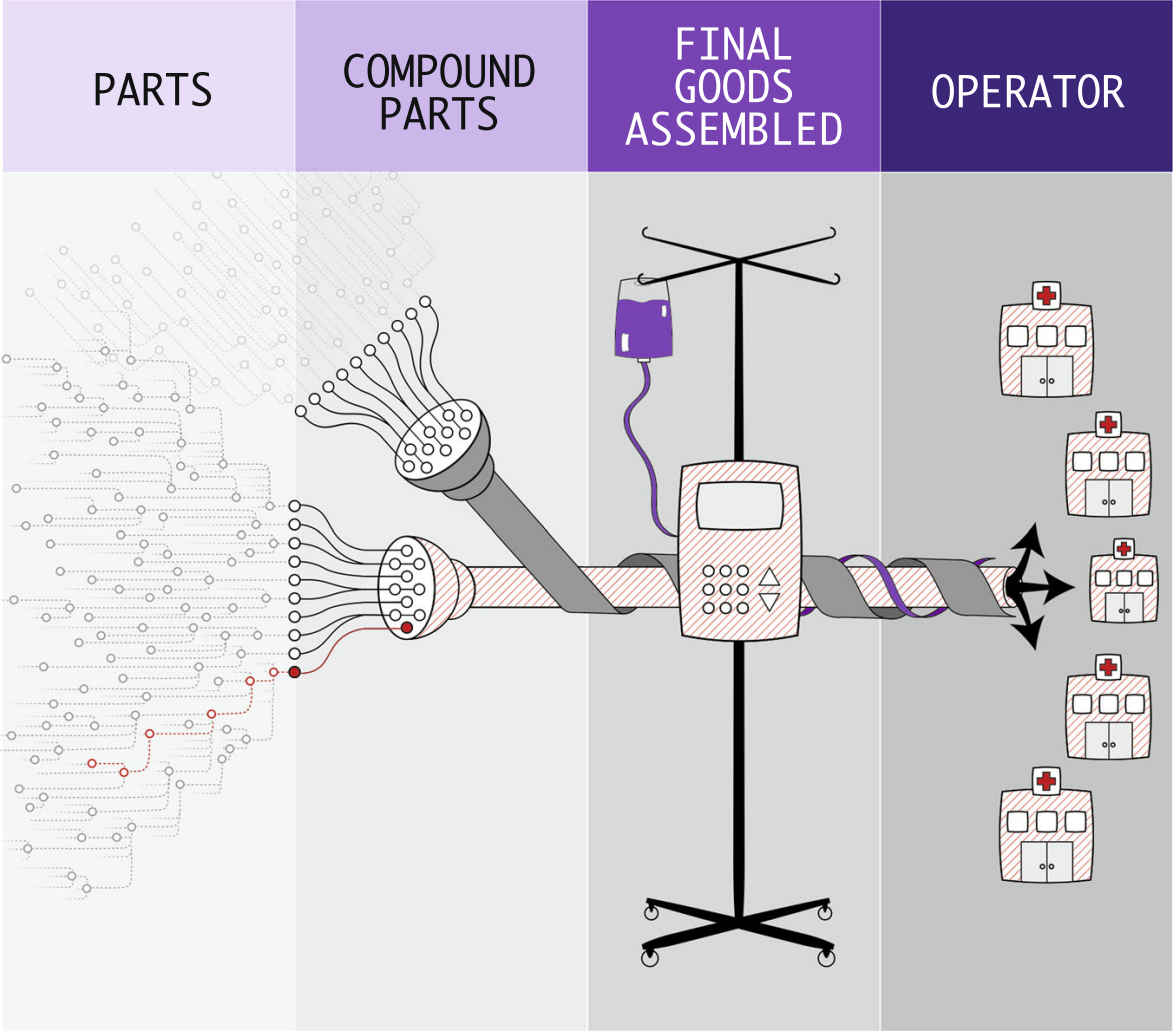
The software supply chain ecosystem. The software supply chain ecosystem consists of manufacturers of parts, compound parts, and final goods assembled, and operators. A software bill of materials provides visibility into the contents of software throughout the supply chain. Reprinted from NTIA Use Cases and State of Practice Working Group^4^.

Software vulnerabilities and proof-of-concept exploits are often publicly known before they are used by adversaries. SBOMs are a tool that allows stakeholders to better manage cost and risk, both individually and across the healthcare ecosystem, by revealing the presence of vulnerable software components to various supply-chain stakeholders. These stakeholder roles include the builder (developer/manufacturer), buyer (customer), operator (hospital, doctor, and patient), and regulator of software products (see Fig. 2)^4^. Stakeholders can hold multiple roles; for example, a medical device manufacturer (MDM) can be both a builder and buyer of software.

### For the builder

Modern software is composed of both third-party components and custom code. Components and code are updated to improve functionality and to fix software bugs, some of which are security related. An SBOM can make the task of understanding what is included in the build, and therefore what needs maintenance, a more routine process for builders and other supply-chain stakeholders. Removing unneeded components in the final product is also a best practice that reduces the “attack surface” of the application^36^. As already mentioned, the financial services industry uses SBOMs to increase agility, efficiency, and effectiveness in maintaining its software.

### For the buyer

For buyers, an SBOM helps evaluate risk at the time of purchase of a builder’s product (e.g., when an HDO buys a medical device from an MDM). An SBOM reveals individual software component versions that can then be matched to publicly known vulnerabilities, such as those listed in the National Vulnerability Database (nvd.nist.gov). Buyers can also compare different products, evaluating their relative complexity, composition, and quality.

Equipped with this information, buyers can better account for cost and risk in their buying decisions, selecting the best market option for themselves. While the builder may be willing to accept the risk these vulnerable components present to their organization, a buyer may deem the risks—or the costs to mitigate them—unacceptable. Some buyers that require SBOMs from builders might identify components for which they cannot mitigate risks at an acceptable cost. As a result, these buyers can prevent these components, often referred to as “non-permitted technologies”, from entering their environments^32^.

### For operators

**Fig. 3:**
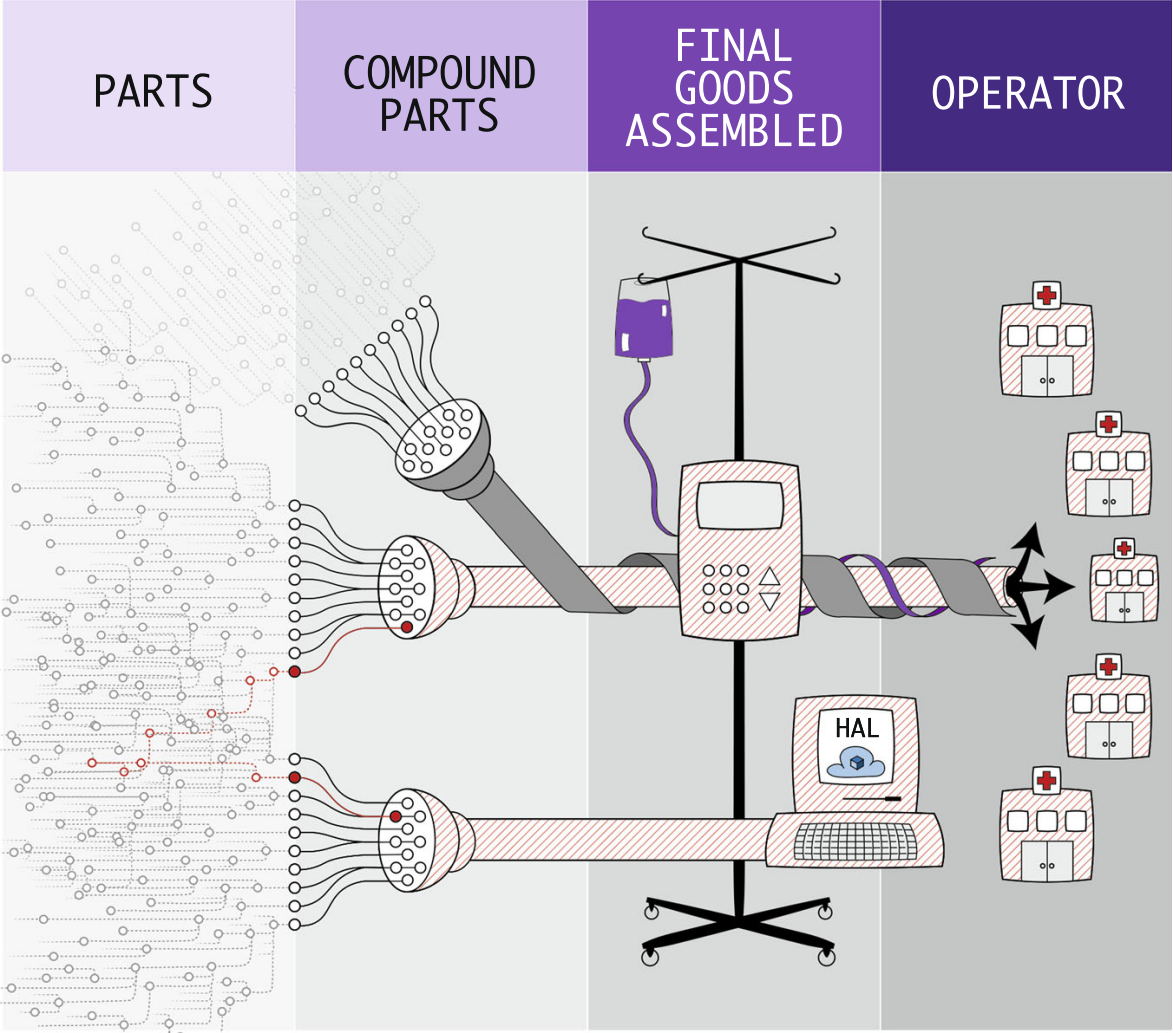
Multiple vulnerability pathways. A single vulnerability has the potential to impact operations via multiple pathways. The same vulnerable third-party component can exist in medical devices and in enterprise systems. Both must be addressed to protect the entire healthcare technology ecosystem. Reprinted from NTIA Use Cases and State of Practice Working Group^4^.

For organizations and individuals who operate and maintain software, tracking an SBOM throughout the product’s lifecycle allows a more proactive security posture by enabling operators to address newly discovered vulnerabilities before adversaries have a chance to compromise them. Traditionally, operators uncover vulnerabilities via point-in-time assessments, penetration tests, or coordinated disclosure notifications. While these methods are necessary to identify certain classes of exposure, they are costly, prone to errors, too slow to keep pace with adversaries, and disruptive to operations, including healthcare delivery. For rapid triage, an up-to-date set of SBOMs can be safely, easily, quickly, and inexpensively mined to understand if and how an organization is impacted by a newly discovered vulnerability (see Fig. 3)^4^. Further, with automation, SBOMs can support continuous vigilance and prompt notification of new, known vulnerabilities that may affect an organization.

### For regulators

For regulators, SBOMs provide a map of overall public health risk when a vulnerability is reported. Analysis of SBOMs across products, companies, and hospitals can reveal and assist in managing systemic risk that would not be apparent within the scope of a single entity. This would also allow regulators to act quickly to reduce potential harm across the healthcare public health sector in the face of newly discovered vulnerabilities. Further, governmental and industry bodies can track vulnerabilities in products that are no longer supported—or whose manufacturers have gone out of business—to continue monitoring for potential security risks.

## Implementing a software bill of materials

Multiple open-source and commercial tools can help builders compile, build, and maintain SBOMs. Many development environments can optionally produce SBOMs at the time the software is compiled^37^. Some code-repository tools monitor component dependencies^38^, provide alerts for security issues in dependencies^39^, or even automatically replace vulnerable dependencies with less vulnerable alternatives^40^. Additionally, some standalone tools offer similar features to those mentioned above^41,42^. Another tool that buyer/operators can leverage for communicating SBOM information is the Manufacturer Disclosure Statement for Medical Device Security, which was updated in October 2019 to include a new SBOM section that “supports controls in the Roadmap for Third Party Components in the Device Life Cycle (RDMP) section”^43^.

While progress has been made in the operationalization of SBOMs, challenges remain. The National Telecommunications and Information Administration (NTIA) multi-stakeholder process on software component transparency is developing industry-led voluntary guidance on standardized formats, use cases, and SBOM tools^44^. Importantly, multinational companies^45^, Philips Medical^46^, and Siemens Healthineers^47^ have pioneered delivery of SBOMs to their customers, putting theory into practice.

## Government and software bill of materials

“The health care system cannot deliver effective and safe care without deeper digital connectivity. If the health care system is connected, but insecure, this connectivity could betray patient safety, subjecting them to unnecessary risk and forcing them to pay unaffordable personal costs. Our nation must find a way to prevent our patients from being forced to choose between connectivity and security.”^48^

In June 2017, the US Health and Human Services (HHS) Cybersecurity Task Force made recommendations to help address cybersecurity within the healthcare public health sector, claiming that healthcare cybersecurity is in critical condition, citing a number of root causes related to software supply chain vulnerabilities^48^. The report outlines how SBOMs could positively impact healthcare and recommended that manufacturers and developers create SBOMs. In November 2017, the Chair and Ranking Member of the US House Committee on Energy and Commerce called on HHS to “convene a sector-wide effort to develop a plan of action for creating, deploying, and leveraging BOMs for health care technologies”^49^. Additional support for the adoption of SBOMs in healthcare came in a May 2018 letter from the Executive Vice President of the American Medical Association to the Energy and Commerce committee^50^.

In April 2018, the US Food and Drug Administration’s (FDA) Center for Devices and Radiological Health (CDRH) released its Medical Device Safety Action Plan^51^. The action plan stated that the FDA was revising their 2014 premarket cybersecurity guidance^52^ and exploring additional authorities that would require an SBOM to be submitted to the FDA prior to a device reaching the market. In October 2018, FDA CDRH released its draft premarket cybersecurity guidance^53^, which stated that leveraging an SBOM may support compliance with federal purchasing controls (21 CFR 820.50)^54^. Purchasing controls could be a significant legal lever for MDMs, as buyers of software, to perform due diligence on software component builders before incorporation into devices. The guidance also states that, as part of risk management, MDMs should provide a bill of materials cross referenced with the National Vulnerability Database or similar known vulnerabilities database. Due to legal compliance, tying quality system regulations for risk management to SBOM could help facilitate the management of third-party component security risk after the device is marketed, including incentivizing changes to devices for cybersecurity. Further, since 2005, FDA’s policy^55^ has stated that changes to devices for cybersecurity typically do not need to be submitted to the agency for clearance or approval (sometimes referred to as recertification)^56–58^, and that not all software changes are recalls^59^.

While HHS and FDA represent significant regulatory incentives for builders and buyers, other governmental efforts have been impactful. In September 2018, the NTIA launched a multi-stakeholder process for software transparency. The NTIA initiative included an SBOM proof-of-concept study for healthcare public health, which was led by MDMs and healthcare delivery organizations^60^. The Joint Commission and Centers for Medicare and Medicaid Services are designated to improve and impress the safety of health information technology for compliance, management, and organizational management. The Department of Defense Risk Management Framework also aligns the organizational and traditional baseline control-selection approaches for a more secure system that holds supply chain vendors and engineers liable for their processes, with a distinct focus on the systems security recommendations from the National Institute of Standards and Technology (NIST)^61^.

Internationally, the International Medical Device Regulators Forum (IMDRF) has published a draft set of principles and practices for medical device security that includes SBOM^62^, as do the Health Canada requirements for medical device security^63^ and EU guidance^64^.

## The path forward

SBOMs have a role to play in further advancing the public’s trust in connected technologies. An SBOM reveals distinctions among products, allows buyers to better account for total cost and risk, and gives buyers better tools to identify, respond to, and recover from vulnerabilities and their effects.

Widespread adoption of SBOM could allow for earlier identification of software vulnerabilities, shorter time to remediation, and heightened awareness of outbreaks and their effects^31^. A growing number of regulators, builders, and operators are recognizing the value of SBOMs. All signs point to SBOM being more widely adopted in the coming years, particularly in industries where technology is life-critical and transparency is paramount. The rate of adoption will increase if the efforts outlined in this paper continue to move forward. Increasing transparency unlocks and enables trustworthy, resilient, and safer healthcare technologies for all.
